# TESTING OF A WEB-BASED DECISION AID TOOL FOR DEMENTIA CARE PLANNING AMONG CHINESE AMERICAN FAMILY CAREGIVERS

**DOI:** 10.1093/geroni/igad104.3623

**Published:** 2023-12-21

**Authors:** Chien-Ching Li, Xiao Ying Lei, Olimpia Paun

**Affiliations:** Rush University, Chicago, Illinois, United States; Rush University, Chicago, Illinois, United States; Rush University, Chicago, Illinois, United States

## Abstract

In the US, the Chinese American population is aging rapidly. It is anticipated that Chinese American family caregivers of loved ones living with dementia will face increasing needs for health care planning. The study aimed to examine the usability and acceptability of a linguistically and culturally appropriate and wen-based decision aid (DA) tool for home care and long-term care planning among Chinese American family caregivers. The DA tool provides caregivers with information on (1) dementia stages, behaviors, symptoms, and care tips, (2) home care and long-term care choices, and their benefits and risks, (3) what matters to you when making care decisions, (4) what else do you need to make your decision, and (5) a summary to facilitating decision making with family and providers. A total of 16 family caregivers were recruited in the study. About 88% of study participants were very satisfied with the DA tool. Key feedback reported by the majority of participants includes: (1) the length and amount of information presented in the tool was just right, (2) this tool can provide sufficient information to make appropriate care decisions for loved ones with dementia., (3) the tool is easy to use and most people would learn to use this tool very quickly, (4) various functions in this tool are well integrated, and (5) I will frequently use this tool to help me discuss dementia care plans and decisions with family and providers. Further intervention with the DA tool can be developed within the Chinese population.

